# TDRD6 mediates early steps of spliceosome maturation in primary spermatocytes

**DOI:** 10.1371/journal.pgen.1006660

**Published:** 2017-03-06

**Authors:** Müge Akpınar, Mathias Lesche, Grigorios Fanourgakis, Jun Fu, Konstantinos Anasstasiadis, Andreas Dahl, Rolf Jessberger

**Affiliations:** 1 Institute of Physiological Chemistry, Medical Faculty Carl Gustav Carus, Technische Universität Dresden, Dresden, Germany; 2 Deep Sequencing Group SFB 655, Biotechnology Center, Technische Universität Dresden, Dresden, Germany; 3 Stem Cell Engineering, Biotechnology Center, Technische Universität Dresden, Dresden, Germany; Cornell University, UNITED STATES

## Abstract

Tudor containing protein 6 (TDRD6) is a male germ line-specific protein essential for chromatoid body (ChB) structure, elongated spermatid development and male fertility. Here we show that in meiotic prophase I spermatocytes TDRD6 interacts with the key protein arginine methyl transferase PRMT5, which supports splicing. TDRD6 also associates with spliceosomal core protein SmB in the absence of RNA and in an arginine methylation dependent manner. In *Tdrd6*^*-/-*^ diplotene spermatocytes PRMT5 association with SmB and arginine dimethylation of SmB are much reduced. TDRD6 deficiency impairs the assembly of spliceosomes, which feature 3.5-fold increased levels of U5 snRNPs. In the nucleus, these deficiencies in spliceosome maturation correlate with decreased numbers of SMN-positive bodies and Cajal bodies involved in nuclear snRNP maturation. Transcriptome analysis of TDRD6-deficient diplotene spermatocytes revealed high numbers of splicing defects such as aberrant usage of intron and exons as well as aberrant representation of splice junctions. Together, this study demonstrates a novel function of TDRD6 in spliceosome maturation and mRNA splicing in prophase I spermatocytes.

## Author summary

Very little is known about splicing and its regulation in germ cells, particularly during meiosis. This paper describes the role of a male germ cell-specific protein, Tudor containing protein 6 (TDRD6), in assembly of the spliceosome in spermatocytes. We show that in prophase I TDRD6 interacts with the key protein methyltransferase of the splicing pathway PRMT5. PRMT5 methylates arginines in substrate proteins. In a methylation dependent manner, TDRD6 also associates with spliceosomal core protein SmB in the absence of RNA, thus before an RNP-type spliceosome has been assembled. In *Tdrd6*^*-/-*^ diplotene spermatocytes PRMT5’s association with SmB and arginine dimethylation of SmB are much reduced. Abrogation of arginine methylation impairs the assembly of spliceosomes and the presence of the spliceosomal RNA U5 is aberrantly increased. These deficiencies in spliceosome maturation correlate with decreased numbers of Cajal bodies and gems involved in later stages, i.e. nuclear snRNP maturation. To reveal functional consequences of these deficiencies, transcriptome analysis of primary spermatocytes showed high numbers of splicing defects such as aberrant usage of intron and exons as well as aberrant representation of splice junctions upon TDRD6 loss. This study reveals a novel function of TDRD6 in spliceosome maturation and mRNA splicing in spermatocytes

## Introduction

Spermatogenesis is essential for the generation of haploid male gametes required for sexual reproduction in higher eukaryotes. Spermatogenesis in mice starts at app. day 6 postpartum (dpp) as spermatogonia undergo mitotic expansion, generate tetraploid cells (4N) during premeiotic S-phase and enter meiosis. Meiosis is composed of two successive nuclear divisions. In the first meiotic division (meiosis I), pairs of homologous chromosomes are segregated and primary spermatocytes are reduced in chromosome content to diploid secondary spermatocytes (2N). These 2N cells then undergo reduction to haploid spermatids (N) in the second meiotic division (meiosis II) through a mitosis-like division segregation of sister chromatids. Meiotic prophase I is by far the longest phase of meiosis, lasting approximately three weeks in most mammals. It is described by four sequential substages, i.e. leptotene, zygotene, pachytene and diplotene. Prophase I features unique chromosome properties and behavior such as the pairing of homologous chromosomes and formation of the synaptonemal complex (SC) in pachytene. The SC consists of two axial elements (AE) that form earlier during leptotene. Each AE–often visualized by staining for protein SYCP3 –supports the two sister chromatids of one homologue. In the diplotene stage, homologues desynapse and remain linked only at the sites of crossing-over/chiasmata which are resolved at metaphase/anaphase (reviewed in [1]). Two successive meiotic divisions follow prophase I to produce haploid spermatids leading to the last stage of spermatogenesis called spermiogenesis; i.e. the process of morphological differentiation of haploid round spermatids to elongated spermatids to motile sperm.

Chromatin compaction during the late steps of spermatogenesis results in silencing of transcription at this stage despite ongoing translation of mRNA. This is enabled through the temporal uncoupling of transcription and translation of mRNA during spermatogenesis [2]. Most mRNA is transcribed at earlier stages, i.e. late prophase I (late pachytene, diplotene) and early spermiogenesis (reviewed in [3]). Direct measurement of *de novo* synthesized RNA during spermatogenesis demonstrates a peak in global transcription in late prophase I, which meets the later demand and provides the mRNA needed at this stage [4–7]. This entails posttranslational regulation and storage of these mRNAs (reviewed in [8, 9]). Germ cells are endowed with special granules involved primarily in posttranscriptional regulation of mRNA. These granules include the fibrous-granular chromatoid body (ChB) type 1 in late prophase I and the single globular ChB type 2 in round spermatids (reviewed in [10, 11]).

The ChBs contain mRNA, miRNA and piRNA as well as proteins functioning in RNA metabolism such as the DEAD-box RNA helicase MVH [12–14]. Tudor domain (TDRD) containing proteins are prominent components of ChBs. The tudor domain is a conserved protein structural motif of ~60 amino acids and was initially found in proteins that associate with nucleic acids [15]. It is characterized by a barrel-like structure composed of anti-parallel ß-sheets forming a hydrophobic pocket surrounded by charged residues that constitute a protein-protein interaction surface [16–19]. A scaffolding function has been suggested for TDRD proteins [20, 21]. Mammalian TDRD proteins contain variable numbers of Tudor domains that are occasionally accompanied by other types of domains. The Tudor domain requires methylated or di-methylated arginines or lysines for binding target proteins. Among the TDRD proteins in male germ cells, TDRD6 is expressed only from mid prophase I spermatocytes onwards and localizes to the ChBs [20, 22, 23]. Mouse TDRD6 encodes 6 Tudor domains and is the homologue of *Drosophila* Tudor protein. It appears first in late pachytene spermatocytes (day 17.5 post partum) in multiple fine filamentous cytoplasmic granules surrounding the nuclei. In round spermatids, the TDRD6 signal appears as a single bright distinct perinuclear dot representing the ChBs. TDRD6 interacts *in vitro* with MVH, MILI and MIWI and loss of TDRD6 results in mis-localization of ChB components MVH and MIWI. In the absence of TDRD6 the ChB architecture is severely impaired and spermiogenesis is arrested at the round-to-elongated spermatid stage. Pre-miRNA and miRNA transcripts are mis-regulated in *Tdrd6*^*-/-*^ testis [22]. Lately we reported the role of TDRD6 in the long 3’ UTR-stimulated pathway of NMD during spermiogenesis [13]. Overall, these findings implicate that TDRD6 functions in distinct RNA pathways during spermatogenesis, but functions in meiosis remained unclear.

Mass spectrometric analysis of potential TDRD6 associated proteins [22] identified PRMT5, a key protein methyl transferase of the splicing pathway. This suggested a role for TDRD6 in the pre-mRNA splicing pathway. The studies reported here revealed a central role for TDRD6 in the regulation of spliceosome maturation and mRNA splicing in spermatogenesis.

The spliceosome is a large and flexible macromolecular machine with more than a hundred RNA and protein components [24]. Small nuclear ribonucleoproteins (snRNPs) constitute the main building blocks of the spliceosome machinery. Different types of spliceosomal snRNPs all contain a set of seven common Sm proteins (SmB/B’, D1, D2, D3, E, F, G), which form a heptameric ring around spliceosomal snRNAs, commonly referred to as U-RNAs due to their high uridine content. The majority of pre-mRNAs is spliced by the major spliceosome, which contains a combination of U1, U2 or U5 or both U4/U6 snRNAs. A small group of introns called ‘ATAC introns’ are spliced by the minor spliceosome containing a combination of U11, U12, U4atac, U6atac or U5 snRNAs. Formation of the heptameric Sm ring and its assembly with individual U-RNAs is catalyzed by the SMN complex in an ATP-dependent manner *in vivo* although this assembly can also take place spontaneously *in vitro* [24–26]. The SMN complex is composed of the Tudor-domain containing protein SMN, seven Gemin proteins (Gemin 2–8) and unrip. The SMN complex cooperates with the PRMT5 “methylosome” complex, which is comprised of WD45/MEP5, pICln and the protein arginine methyltransferase 5 (PRMT5) [27]. SMN-positive snRNP bodies translocate to the nucleus and are further modified to mature. These modifications take place in a non-membraneous nuclear organelle called Cajal body (CB) [28]. CBs are characterized by the accumulation of coilin, a protein essential for CB integrity, as well as scaRNAs, which are small RNA species transcribed by RNA pol III and targeted specifically to CBs as RNPs [29, 30]. After undergoing these multiple steps of maturation, the spliceosomes are ready to act on the primary mRNA transcripts. Very little is known about spliceosome assembly and regulation of splicing in spermatocytes, and the present study reveals a male germ cell-specific pathway of spliceosome assembly.

## Results and discussion

### TDRD6 interacts with PRMT5

A previous mass spectrometric analysis performed in our lab [22] revealed PRMT5 as a putative interactor of TDRD6. PRMT5 catalyzes the symmetrical arginine dimethylation of proteins including SmB [24, 31, 32], one of the core protein components of spliceosomal snRNPs (reviewed in [33, 34]). PRMT5 belongs to a family of protein arginine methyl transferases that are widely expressed and that di-methylate arginine in an either symmetric (sDMA) or asymmetric (aDMA) manner. PRMT5, a so-called type II arginine methyl transferase, generates sDMA marks (recently reviewed in [35]). PRMT5 associates with Sm proteins [24] and PRMT5 deficient somatic cells display splicing defects [36]. Dart5, the Drosophila ortholog of murine PRMT5 is essential for germ cell specification and maintenance [37]. In mice, PRMT5 is dispensable for primordial germ cell specification. Still, PRMT5 regulates gene expression by altering the spliced repertoire of RNAs as it promotes arginine methylation of the Sm proteins in primordial germ cells [38].

To validate the interaction between TDRD6 and PRMT5, we generated a transgenic mouse carrying a Localization and Affinity Purification ‘‘LAP”- tagged TDRD6 BAC construct [39]. The LAP tag contains an EGFP sequence for localization studies and for immunoprecipitation (IP) (S1A Fig). In immunoblots (IB) of total cell extracts from LAP-TDRD6-positive transgenic testes an α-EGFP antibody recognized two bands of 250 kDa (cleaved LAP-TDRD6) and 270 kDa (full-length LAP-TDRD6), indicating that LAP-TDRD6 is processed like the endogenous protein [22] (S1B Fig). Fluorescent microscope analysis of testis sections of adult transgenic mice demonstrated as expected a fibrous and granulated perinuclear EGFP (LAP-TDRD6) signal in primary spermatocytes of stage X-XI sections (S1Ci Fig) and one single lobulated, perinuclear EGFP signal in round spermatids of stage I-II-III sections (S1Cii Fig). The typical structure of wild-type tubules was also maintained (S1C Fig). Motile spermatozoa were observed in the testis of LAP-TDRD6-positive *Tdrd6*^*-/-*^ males from all founders. All LAP-TDRD6-positive *Tdrd6*^*-/-*^ males were fertile. This proves that the LAP-TDRD6 is fully functional as it can rescue the developmental arrest during spermiogenesis and the sterility of *Tdrd6*^*-/-*^ males.

**Fig 1:**
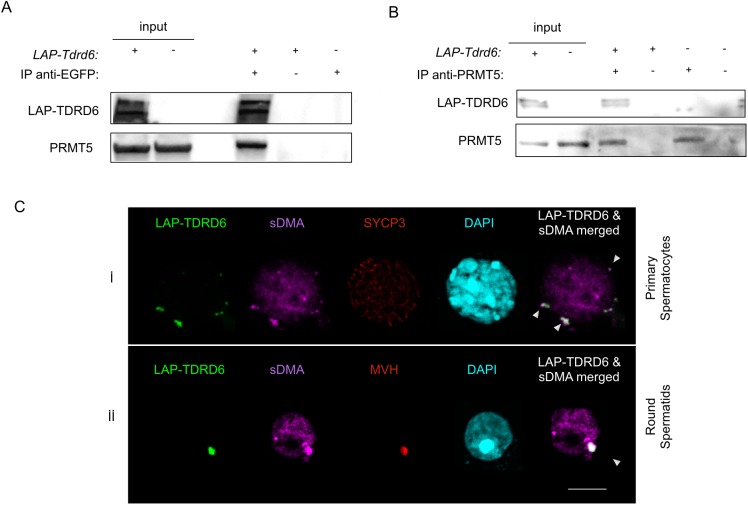
TDRD6 interacts with PRMT5 and it is a hub for symmetrically dimethylated proteins at arginine residues in the cytoplasm of murine male germ cells. **(A)** α-EGFP IgG was used to immunoprecipitate LAP-TDRD6 and **(B)** α-PRMT5 IgG was used to precipitate PRMT5 from total cell extracts of formaldehyde-fixed adult testis cells. Precipitates were separated by SDS-PAGE and protein immunoblot was performed using α-EGFP and α-PRMT5 IgGs. **(C)** Immunofluorescence staining of EGFP-positive adult testis cells with α-SYCP3 (red) or α-MVH (red) and α-SYM (magenta) for sDMA-containing proteins and DAPI (cyan). Cells at different stages of spermatogenesis are indicated. Colocalization of LAP-TDRD6 and sDMA signal is indicated with arrowheads. Scale bar: 5μm.

LAP-TDRD6 was immunoprecipitated with an α-EGFP antibody from RIPA extracts of total adult testes and probing the IB for PRMT5 detected the PRMT5 signal (Fig 1A). IB probing of the reverse IP with an α-PRMT5 antibody yielded LAP-TDRD6, confirming the interaction (Fig 1B).

### TDRD6 interacts with symmetrically dimethylated SmB in the absence of RNA

TDRD6 has 6 Tudor domain [22], which mediate protein-protein interactions by recognizing and binding to symmetrically dimethylated arginine (sDMA) residues generated by methyltransferases like PRMT5 [18]. In primary (pachytene, diplotene) spermatocytes, identified by anti SYCP3 staining, the majority of TDRD6 localizes to filamentous ChB type 1 structures in the perinuclear space (Fig 1C, panel i). In later stages (round, elongated spermatids), the single TDRD6 signal localizes to the ChBs type 2 marked by the MVH signal as reported before [22], which also overlaps with sDMA signals (Fig 1C, panel ii). The lack of sDMA signals in precipitates of LAP-TDRD6 suggests that TDRD6 itself is not symmetrically dimethylated and excludes that the overlap of all TDRD6 with sDMA signal is due to the methylation of TDRD6 itself (S2 Fig). This data is consistent with previous reports showing the association of Tudor domains with proteins carrying sDMA. It appears likely that this interaction contributes also to the architecture of ChBs, which harbor many sDMA-proteins such as MILI, MIWI, UPF1 and UPF2 [12, 13, 22].

**Fig 2:**
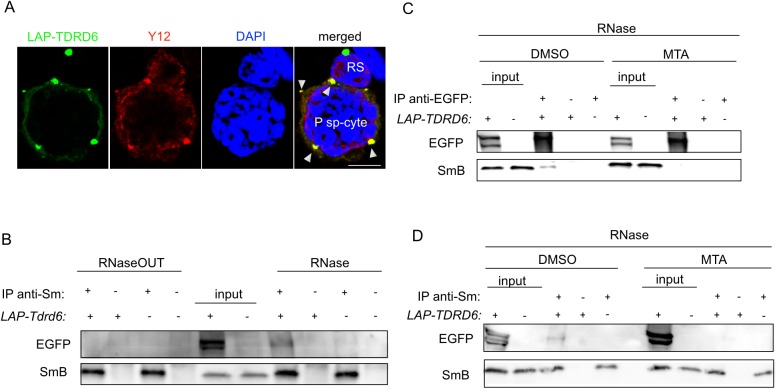
TDRD6 and SmB interact in the perinuclear space of primary spermatocytes in the absence of RNA and in the presence of sDMA. **(A)** Immunofluorescence staining of EGFP-positive adult testis cells with α-Y12 (red) for spliceosomal core proteins and DAPI (blue). Colocalization of LAP-TDRD6 and Sm signals is indicated with arrowheads. P sp-cyte: primary spermatocyte; RS: round spermatid. Scale bar: 5μm. **(B)** α-Y12 IgG was used to immunoprecipitate Sm proteins from total cell extracts of adult testis cells in the presence of RNaseOUT or RNase. Precipitates were separated by SDS-PAGE and immunoblotting was performed using α-EGFP and α-Y12 IgGs. **(C)** α-EGFP or **(D)** α-Y12 IgGs was used to immunoprecipitate LAP-TDRD6 or Sm in the presence of RNAse using total cell extracts of adult testis cells cultured for 16h with MTA or DMSO (control). Precipitates were separated by SDS-PAGE and immunoblotting was performed using α-EGFP and α-Y12 IgGs.

The assembly of spliceosomal proteins with U-RNAs is mediated partly through direct interactions between the Tudor domain of SMN and the sDMAs of the spliceosomal proteins SmB, SmD1, SmD3 [16]. We hypothesized that TDRD6 would associate with sDMA-containing spliceosomal core proteins. Staining of LAP-TDRD6 germ cells with α-Y12 antibody recognizing SmB and SmD revealed an overlap between the Y12 and LAP-TDRD6 signals in the nuclear periphery of diplotene cells but not in the TDRD6-positive ChBs in post-meiotic round spermatids (Fig 2A). The absence of Sm proteins in ChB type 2 was also confirmed by our recent proteomic analysis of ChBs in round spermatids [13].

Sm proteins bind to the U-RNAs in spliceosomes and RNA could bridge its interaction with TDRD6. Therefore, we precipitated Sm proteins with α-Y12 IgG both in the absence and presence of RNase from total testes of adult males. We used SmB detected as a 25 kDa band to represent spliceosomal proteins. The interaction was completely lost in the absence of RNase (Fig 2B). Successful degradation of RNA was confirmed by staining of the total RNA extracted from the flow-through of the IP (S3 Fig). The interaction of TDRD6 with Sm proteins exclusively in absence of RNA places TDRD6 upstream of the association of SmB with the SMN complex during spliceosome maturation, i.e. before U RNA binding to the complex.

We then tested whether the sDMA modification mediates the TDRD6-Sm interaction. We inhibited methylation with 5′-deoxy-5′-(methylthio)adenosine (MTA) in total adult testis cultures for 16 h prior to IP. In the presence of MTA the interaction between LAP-TDRD6 and SmB is undetectable as shown by both α-EGFP (Fig 2C) and the reverse α-Y12 IPs (Fig 2D), while the interaction was observed in control-treated cells. To exclude the possibility that the interaction is lost due to apoptosis upon MTA treatment, we analyzed MTA and control-treated cells after 16 h via FACS by staining them for Annexin V and propidium iodide. There was no significant change in the early or late apoptotic and dead cell populations of MTA versus control cells (S4 Fig). Thus, we conclude that TDRD6 requires sDMA to interact with SmB.

Previous studies in our lab revealed that about 20 kDa off the C-terminus of Tdrd6 are removed during the transition from meiosis I to meiosis II [22]. We can speculate that the app. 20 kDa C-terminal region supports the localization of SmB to ChBs. This may explain the restriction of Sm-TDRD6 co-localization to meiotic stages as detected by immunostaining. The eliminated C-terminal fragment does not contain a Tudor domain as the most C-terminal Tudor domain is at position 1630 of the total of 2135 amino acids. Removal of the C-terminal fragment might affect the 3D-structure of the protein and thereby its interactions.

### SmB interaction with PRMT5 is abrogated and sDMA modification of SmB is diminished in TDRD6-deficienct cells

PRMT5 functions in a complex of cofactors determining its substrate specificity [40–42]. Having shown that TDRD6 interacts with both PRMT5 and one of PRMT5’s substrates, SmB, we hypothesized that TDRD6 regulates methylation of SmB by PRMT5 and that the sDMA status of SmB is altered in *Tdrd6*^*-/-*^ cells. Given that the TDRD6 and SmB signals overlap only in diplotene cells (Fig 2A), we isolated diplotene cells to IP SmB and assess its sDMA status by IB.

Diplotene cells were isolated using anti-hCD4 antibody-coupled magnetic beads binding to germ cells expressing hCD4 under control of the endogenous *Tdrd6* promoter from males at 20 dpp [22]. At this time point the juvenile mice showed no difference in development (S5A Fig) and in apoptosis (S5A and S5B Fig) between *Tdrd6*^*+/-*^ or *Tdrd6*^*-/-*^ strains, and cell preparations containing app. 80% diplotene cells were obtained (S5A Fig).

**Fig 3:**
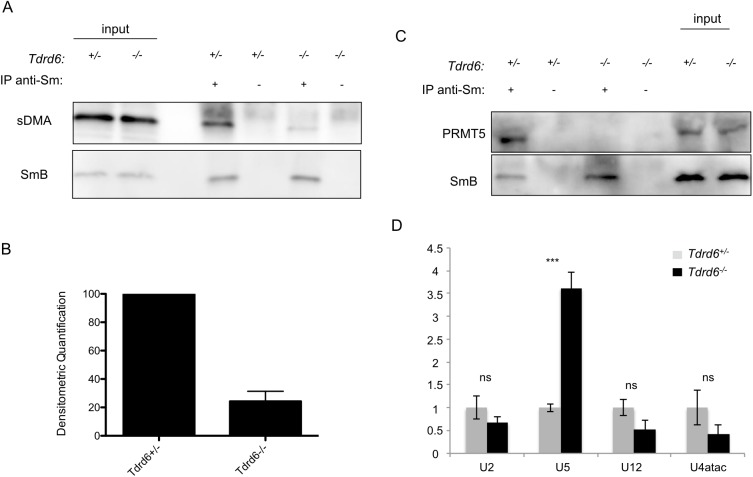
Symmetrical dimethylation of SmB is decreased, its interaction with PRMT5 and the assembly of U snRNPs are impaired in *Tdrd6*^*-/-*^ primary spermatocytes **(A)** α-Y12 IgG was used to immunoprecipitate Sm proteins from total cell extracts of diplotene cells. Precipitates were divided into two and separated by SDS-PAGE in parallel. Immunoblotting was performed using α-Y12 IgG for SmB and α-SYM IgG for sDMA modification. One representative blot of 5 biological repeats is displayed. **(B)** Densitometric quantification of the decrease in sDMA signal on SmB from 5 biological replicates as in (A). **(C)** α-Y12 IgG was used to immunoprecipitate Sm proteins from total cell extracts of formaldehyde-fixed diplotene cells. Precipitates were separated by SDS-PAGE. Western blotting was performed using α-Y12 IgG for SmB and α-PRMT5 IgG. (n = 3 biological replicates) **(D)** RNA immunoprecipitation (RIP) using α-Y12 IgG against Sm proteins, followed by RT-qPCR for U2, U5, U12, U4atac snRNAs. (n = 3 biological replicates, each with 3 technical replicates; *P* = 0.0017 for U5 while *P* > 0.05 for U2, U12 and U4atac)

We IPed SmB and observed a strong decrease of 76% (Fig 3B; SD = ±7.1%) in the sDMA signal on SmB in *Tdrd6*^*-/-*^ diplotene cells (Fig 3A). Considering that TDRD6 and PRMT5, TDRD6 and SmB, as well as PRMT5 and SmB interact with each other, TDRD6 may act as a tissue-specific mediator of PRMT5 for SmB methylation in the testis. TDRD6 may bring PRMT5 and SmB in close vicinity to each other and thereby facilitate the methylation. Alternatively, TDRD6 may interact with another cofactor of PRMT5 and load it on the methylosome complex, thereby indirectly controlling its activity. We analyzed the interaction between the enzyme and its substrate. IPs with α-Y12 revealed a complete loss of PRMT5 co-precipitation with SmB in *Tdrd6*^*-/-*^ diplotene cells (Fig 3C).

An sDMA mark, which needs to be generated by another PRMT, is a known prerequisite for binding of PRMT5 to SmD3 [43]. It is unclear which PRMT modifies PRMT5. PRTM9 would be another type II PRMT with di-methylation activity but is very lowly expressed in germ cells as we observed in our transcriptome data of primary spermatocytes (see below). We detected 3-fold higher expression of PRMT7 than PRMT5 in the transcriptome suggesting that PRMT7 functions in these cells. It is controversial, however, whether PRMT7 only mono-methylates or also di-methylates as has been reported for Sm proteins [43] and other proteins, at least in vitro [44]. Therefore we can only speculate that PRMT7 may account for the remaining methylated pool (24%) of SmB detected in TDRD6-deficient murine testis, and PRMT7 may also be responsible for PRMT5 methylation.

An additional means of contribution by TDRD6 to overall sDMA levels may be protection of sDMA from protein demethylases. While the existence of arginine demethylases is still debated, recent evidence argues in favor of such enzymes [45]. Binding to sDMA on other proteins like PRMT5, TDRD6 may protect sDMA marks. In absence of TDRD6, these sDMA marks–such as those on PRMT5 –may get lost. As a consequence the interaction of PRMT5 with SmB and its methylation by PRMT5 may be affected [43].

### The assembly of spliceosomal snRNPs is impaired upon TDRD6 loss

After methylation, Sm protein sub-complexes are loaded onto the SMN complex, which mediates their assembly with the U-RNAs [46]. In human cells, cytoplasmic snRNP assembly requires the activities of both PRMT5 and PRMT7 [43]. Since the sDMA status of SmB is diminished in *Tdrd6*^*-/-*^ diplotene cells, we hypothesized an impaired assembly of U-snRNPs at this stage. Using the same *Tdrd6*^*+/-*^ and *Tdrd6*^*-/-*^ diplotene stage cell populations, we performed RNA IP with the α-Y12 antibody and compared the levels of selected U-RNAs incorporated into the spliceosomes via real-time RT-PCR. U2 is a component of major spliceosome snRNPs while U12 and U4atac are found in minor spliceosomes. U5 is common in both (reviewed in [33]). We observed a 3.5-fold (SD = ± 0.5, *P* < 0.05) increase in U5 RNA levels in the splicing snRNPs in the absence of TDRD6. There was no significant change in the levels of U2, U12 and U4atac bound to Sm proteins (*P* > 0.05) (Fig 3D). SmB IB of the RNA IPs with α-Y12 IgG confirms the equal pull-down of this protein in both *Tdrd6*^*+/-*^ and *Tdrd6*^*-/-*^ samples (S6 Fig).

This finding together with the requirement for RNA exclusion in SmB-TDRD6 interaction suggests that TDRD6 acts as an assembly factor for at least SmB and either competes with certain snRNAs like U5 for SmB binding or can only bind in an early step of spliceosome maturation prior to RNA binding.

Exclusion of a certain U-RNA by TDRD6 resembles the mode of interaction of pICln with Sm proteins. pICln acts as a chaperone by binding to the small, highly conserved Sm domain that is N-terminal to the methylated RG motifs and responsible for oligomerization and RNA binding of Sm proteins [47]. As elaborated above, TDRD6-SmB binding is likely mediated by Tudor-sDMA interaction. The precise mode and domain requirements for TDRD6-SmB interactions are subject to future studies. We speculate that by binding to SmB at the methylated RG box, TDRD6 may trap sDMA-marked SmB and sterically occlude the assembly with U5. The determinants for this specificity and also for each U-RNA binding to the Sm core are still unknown. U5 may have higher affinity to the Sm core than the other U-RNAs. Alternatively, TDRD6 might suppress a factor responsible for U5 loading on the Sm ring.

### Numbers of both Cajal bodies and SMN-positive nuclear bodies are reduced in *Tdrd6*^*-/-*^ primary spermatocytes

Once the U-snRNP assembly takes place in the cytoplasm, the SMN complex translocates into the nucleus to carry the snRNPs to Cajal Bodies (CBs), where the final steps of spliceosome maturation take place. The rate of spliceosomal snRNP assembly correlates with CB number [48] and SMN depletion results in a decrease in CB abundance [48, 49].

**Fig 4:**
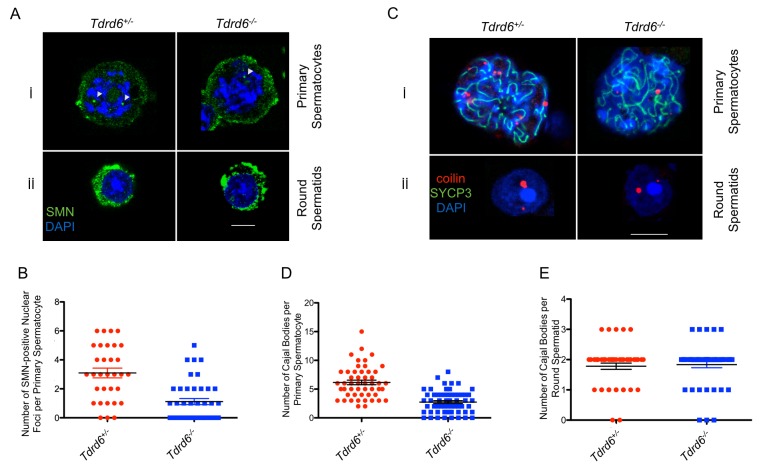
Numbers of SMN-positive nuclear bodies and Cajal bodies (CBs) are diminished upon TDRD6 deficiency. **(A)** Immunofluorescence staining of MACS-isolated hCD4^+^ cells from the testes of *Tdrd6*^*+/-*^ and *Tdrd6*^*-/-*^ littermates with α-SMN (green) and DAPI (blue). Cells at different stages of spermatogenesis are indicated. Scale bar: 5μm **(B)** Quantification of the number of SMN-positive nuclear bodies in primary spermatocytes is presented in a scatter plot. The number of SMN-positive nuclear bodies is reduced by circa 70% upon TDRD6 loss (*P* < 0.0001). (n = 32 for *Tdrd6*^*+/-*^, n = 42 for *Tdrd6*^*-/-*^ derived from 3 different biological replicates) **(C)** Immunofluorescence staining of MACS-isolated hCD4^+^ cells from the testes of *Tdrd6*^*+/-*^ and *Tdrd6*^*-/-*^ littermates with α-coilin (red) and α-SYCP3 IgG (green) and DAPI (blue). Cells at different stages of spermatogenesis are indicated. Scale bar: 5μm **(D)** Quantification of the number of CBs in primary spermatocytes based on the coilin staining in (C) is presented in a scatter plot. The number of CBs is reduced by 50% upon TDRD6 loss (*P* < 0.0001). (n = 50 for *Tdrd6*^*+/-*^, n = 59 for *Tdrd6*^*-/-*^ derived from 4 different biological replicates) **(E)** Quantification of the number of CBs in round spermatids based on the coilin staining in (C) is presented in a scatter plot. The number of CBs did not change upon TDRD6 loss (*P* = 0.7245). (n = 46 for *Tdrd6*^*+/-*^, n = 48 for *Tdrd6*^*-/-*^ from 4 biological replicates)

Given the disrupted U-snRNP assembly in *Tdrd6*^*-/-*^ diplotene cells, we hypothesized that formation of both nuclear SMN-positive bodies and CBs would be impaired. Via immunofluorescence staining, we detected a significant decrease by almost 70% in the number of SMN-positive bodies per nucleus (Fig 4B, *p*<0.0001) with a median number of 3 per *Tdrd6*^*+/-*^ nucleus *versus* 1 in *Tdrd6*^*-/-*^ (Fig 4Ai). There were no SMN-positive nuclear bodies detected in round spermatids (Fig 4Aii)

In *Tdrd6*^*+/-*^ diplotene cells, the number of CBs was reduced by 50% (Fig 4D; *p*<0.0001) with a median number of 6 bodies in *Tdrd6*^*+/-*^ *versus* 3 in *Tdrd6*^*-/-*^ (Fig 4Ci). In round spermatids the median number of CBs in round spermatids remained 2 in both genotypes (Fig 4Cii & 4E; *p* = 0.7245). Higher numbers of both SMN-positive bodies and CBs in meiotic cells correlates with the previous data on the increased mRNA transcription and hence splicing in late prophase I [4].

### TDRD6-deficient primary spermatocytes demonstrate widespread splicing perturbations

Several studies on somatic cells previously revealed improper pre-mRNA splicing upon disruptions at the upstream steps of the spliceosome maturation pathway [50–52]. Our data implicated changes in the snRNP profile of TDRD6-deficient diplotene cells by altering the normal proportion of endogenous snRNPs. Therefore, we hypothesized that TDRD6 deficiency would lead to impaired splicing in diplotene cells.

To test this hypothesis, we performed high-throughput sequencing of total RNA from purified diplotene cells of *Tdrd6*^*+/-*^ and *Tdrd6*^*-/-*^ mice at a depth of 47–51 million paired-end fragments per sample. After aligning the fragments to mouse reference genome (mm10) with GSNAP and annotating them with Ensembl v75, we acquired over 360 million RNA sequence reads in total with a very high unique mappability of more than 90% for each sample (S7 Fig).

**Fig 5:**
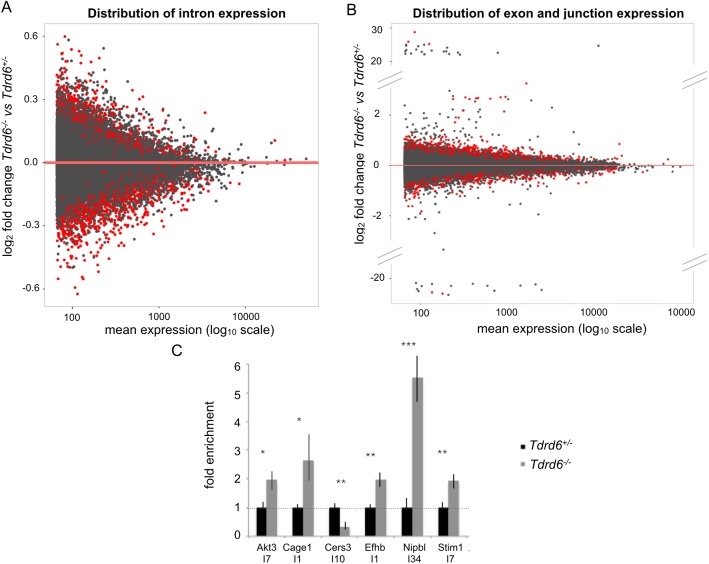
The differential expression of introns, exons and splice junctions in TDRD6-deficient primary spermatocytes. **(A)** Distribution of differential usage of introns in *Tdrd6*^*-/-*^ vs. *Tdrd6*^*+/-*^ primary spermatocytes. Each dot represents one unique intron. The log_2_ fold change in *Tdrd6*^*-/-*^ versus *Tdrd6*^*+/-*^ is represented on the y-axis while log_10_ mean expression for individual introns is displayed on the x-axis. Introns that differ significantly (p-value < 0.1) between the two conditions are represented with red dots. **(B)** Distribution of differentially used exons and exon-exon junctions in *Tdrd6*^*-/-*^ vs. *Tdrd6*^*+/-*^ primary spermatocytes. Each dot represents an event; i.e. one exon or one splice junction. The log_2_ fold change in *Tdrd6*^*-/-*^ versus *Tdrd6*^*+/-*^ is represented on the y-axis while log_10_ mean expression for individual events is displayed on the x-axis. Events that differ significantly (p-value < 0.1) between the two conditions are represented with red dots. **(C)** RT-qPCR of total RNA of *Tdrd6*^*+/-*^ and *Tdrd6*^*-/-*^ diplotene cells using specific primers for I7 of *Akt3*, I1 of *Cage1*, I10 of *Cers3*, I1 of *Efhb*, I34 of *Nipbl* and I7 of *Stim1*. RNA samples were different than those used for deep sequencing (n = 3 technical replicates); * significant at p value<0.1, ** significant at p value<0.05, *** significant at p value<0.01.

We analyzed the splicing profiles using two different approaches. Differential intron usage analysis was performed via DEXSeq with a false discovery rate (FDR) of 0.1. This analysis revealed 1270 genes (of a total of around 20 k genes with detectable expression) with 1850 mis-regulated introns, i.e. 1.5 mis-regulated introns per gene on average (Fig 5A). More specifically, 824 introns showed up-regulation, i.e. retention, in the *Tdrd6*^*-/-*^ samples, whereas 1026 introns showed significant down-regulation compared to the *Tdrd6*^*+/-*^.

Another approach to assess the fidelity of splicing was to test the differential exon and splice site usage. The reads at the exon-exon junctions as well as within the exonic sequences were compared using JunctionSeq. 889 genes demonstrated significant differential usage in 1,989 events indicating more than 2 perturbations at exon or junction level per gene (Fig 5B; FDR = 0.1). 1,509 of these events are exonic and of those, 863 were up-regulated in the *Tdrd6*^*-/-*^ condition while 646 of them were down-regulated. The remaining 471 events represented significantly mis-regulated splice sites. 381 of them were up-regulated while 90 of them showed down-regulation in *Tdrd6*^*-/-*^ primary spermatocytes. These two approaches yielded 240 common genes with mis-spliced mRNA.

**Fig 6:**
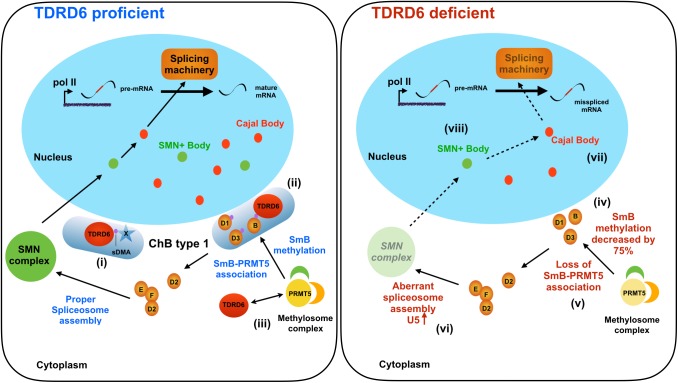
Proposed functions of TDRD6 in primary spermatocyte spliceosome assembly. Shown are primary spermatocytes that are TDRD6 proficient (left) or deficient (right). TDRD6 resides in the chromatoid body (ChB) type 1 of TDRD6 proficient primary spermatocytes and associates with sDMA-modified proteins (represented by blue star-shaped protein X; the violet dot indicates sDMA) (i) such as sDMA-containing SmB, a member of the Sm protein group (B, D1, D2, D3, E, F) (ii). TDRD6 and PRMT5 interact (iii). In TDRD6 deficient primary spermatocytes, no ChB type I form, PRMT5 association with SmB is impaired, (iv) and symmetrical dimethylation of SmB is decreased by 75% (v). U5 spliceosome assembly is upregulated compared to TDRD6 proficient cells and an aberrant SMN complex forms (vi). In the nucleus, the numbers of Cajal bodies (vii) and SMN-positive (SMN+) bodies (viii) are decreased upon TDRD6 deficiency. The names of all proteins and cellular structures are indicated; sDMA, symmetrically dimethylated arginine.

We validated a subset of these common 240 mis-spliced mRNA found by both approaches via RT-qPCR. Genes with several isoforms were excluded since different isoforms may account for the differential usage of introns, exons or junctions detected by these different approaches. We detected the mis-regulation of an intron by designing primers such that one primer resides in the mis-regulated intron whereas the other primer resides in the adjacent unchanged exon to ensure that any difference revealed by PCR would be due to the mis-spliced intron. This way we also avoided unknown genes perhaps expressed within the introns, and accounted for the differences that could arise from differential expression of putative gene(s) lying within that intron (S7B Fig). Overall, we validated mis-regulated introns in 6 different randomly selected genes by RT-qPCR (Fig 6C).

Our data demonstrates slightly less intron retention (824 introns) than intron removal (1026 introns) among the mis-regulated cases. This suggests that upon TDRD6 loss the specificity of the intronic splicing site selection is affected more than the overall efficiency of splicing. This is plausible considering that loss of TDRD6 leads to an altered snRNP repertoire. U5 snRNP is a common component of both types of splicing machineries and therefore centrally important [33]. Increased levels of one type of core snRNP of the splicing machinery might disturb the correct stoichiometry of splicing machinery components.

Functional cluster analysis of the mis-spliced transcripts did not reveal any significantly enriched clustering of these transcripts at a particular cellular function. Also, the mis-regulated miRNAs reported earlier in TDRD6 deficient spermatids [22] are not overrepresented among the 1270 genes showing mis-regulated introns.

The finding that a wide diversity of genes was affected indicates a global role of TDRD6 in the regulation of pre-mRNA splicing.

## Conclusions

The data reported here suggest a novel function for TDRD6 in the maturation of spliceosomal snRNPs during the transcriptionally highly active prophase I of spermatogenesis. This function adds to the roles of TDRD6 at later stages of spermatogenesis/spermiogenesis in formation of the ChB type 2 and in nonsense-mediated decay. One notable difference between TDRD6 protein in meiosis I and in later stages is the conversion of the early 250 kDa form into the later 230 kDa form, which lacks about 20 kDa off its C-terminus We speculate that the 250 kDa variant is the one acting in spliceosome assembly while the 230 kDa variant–the only form present after meiosis I–acts in ChB type 2 processes. It will be fascinating in the future to assign specific molecular properties to the C-terminal end of TDRD6, which does not bear Tudor domains, and *in silico* analysis of the amino acid sequence did not reveal prominent motifs.

For the role of TDRD6 in spliceosome assembly, we propose the model shown in Fig 6. TDRD6 promotes PRMT5 association with and methylation of SmB and likely other Sm proteins. To do so, TDRD6 itself also associates with both proteins. Thereby TDRD6 may also control SmB availability for SMN complex formation. The SmB methylation and/or its TDRD6 association is required to ensure proper composition of the SMN RNP complex and subsequently for presence of SMN-positive bodies as well as Cajal body formation in the nucleus. In absence of TDRD6, ChB type 1 do not form SmB methylation is largely decreased, SmB cannot be properly regulated by TDRD6 and spliceosome assembly becomes unbalanced with aberrantly overrepresented U5 snRNP and possibly improper composition of other components. This leads to substantially reduced SMN+ bodies and Cajal bodies in the nucleus and to aberrant splicing.

Why is TDRD6 expressed only in the male germ line and even here only from mid-pachytene onwards? Obviously, TDRD6 is not needed for the universal splicing apparatus found in almost all animal cells, and it is not even needed for oocytes at any stage. There is no definitive answer to this question, but there are no ChB in non-male germ cells or somatic cells. Other bodies or speckles harbor splicing factors in those cells, and some like Cajal bodies are shared between spermatocytes and other cell types. Cajal bodies are reduced in absence of TDRD6 because their assembly depends on prior successful assembly of SMN complexes. Late spermatocytes and spermatids are unique in that they stepwise change their chromatin organisation until the genome is packaged into a protamine-dense body that is largely transcriptionally silenced. Thus the need to store some RNA species in ChBs, i.e. ChB type 2. There may be testis-specific splice events to produce the appropriate RNA molecules and these are not produced in any other cell type, perhaps explaining the need for TDRD6. However, we find a general splicing deficiency in absence of TDRD6, not only impaired splicing of testis-specific RNAs. Still, TDRD6 may have acquired the role of a spliceosome assembly factor to make sure that all RNA splicing events, including those specific for spermatogenesis, are properly carried out. An additional challenge to spermatocytes stems from the great burst of transcription that happens when chromosome pairing is complete, i.e. when the SCs are formed in pachytene. To handle this burst, which includes testis-specific transcripts, a highly efficient splicing machinery may be required and TDRD6 helps providing that efficiency. Since little is known about splicing processes during mammalian spermatogenesis, this holds intriguing questions for future research.

## Materials and methods

### Animals and ethics statement

Generation of *Tdrd6*^*-/-*^ mice was described in detail previously [22]. *Tdrd6-LAP* mice were generated as described in the Supplemental Experimental Procedures. The use of mice was approved by the State of Saxony animal welfare officials, Az DD24-5131/ 339/6 and was performed according to the national and EU guidelines.

### Antibodies

Antibodies used in several different applications in this study are listed in Tables 1, 2 and 3 in “Supplementary Experimental Procedures”.

### Immunofluorescence for single cell suspensions

Coverslips were boiled in 1M HCl, rinsed with water, dipped into poly-L-lysine solution (50μg/ml poly-L-lysine and 10mM Tris pH8.0 in H_2_O) for 10min at room temperature (RT) and dried at RT. Cells were incubated on coverslips for 1h at RT, fixed in 2% formaldehyde/PBS. From permeabilization/blocking onwards, the same staining protocol was followed as for the sections (see Supplemental Experimental Procedures).

Z-stacks of the single cell suspension stainings were taken with the Leica SP 5 confocal laser-scanning microscope. Orthogonal sections of the images were later obtained using the software Fiji.

### Culturing of testis cells in leptomycin B (LMB) and 5′-Deoxy-5′-(methylthio)adenosine (MTA)

Single cell suspensions were obtained as described in Supplemental Experimental Procedures and incubated at 32°C in Dulbecco's Modified Eagle Medium supplemented with 10% fetal calf serum with 40μg/ml leptomycin B or in 1mM 5′-Deoxy-5′-(methylthio)adenosine (MTA) for 16h. Control cultures were incubated with 70% (v/v) methanol/water for leptomycin B or with DMSO for MTA.

### Immunoprecipitations

100–300μg RIPA extract (see Suppl Exp procedures) was diluted at a 1:2 ratio in IP buffer (50mM Tris-HCl at pH 7.4, 150mM NaCl, 0.25% Triton-X100, 1mM EDTA, 5mM NaF, 1mM Na_2_VO_3_, 1mM PMSF, 1x protease inhibitor cocktail EDTA-free mini), supplemented with the primary antibody and incubated at 4°C overnight with gentle shaking. 8μl Dynabeads-proteinA/G per 1μg of primary antibody was added and incubated at 4°C with gentle shaking for 2h. Beads were washed 3 times for 10 min with the same IP buffer, resuspended in 2x Laemmli buffer and boiled at 95°C for 5 min to elute the proteins. SDS-PAGE and immunoblotting procedures were followed as described in Supplemental Experimental Procedures. Wherever indicated, 100μg/ml RNase or 1U/μl RNaseOUT was added into the RIPA and IP buffers.

Immunoprecipitation for the interactions SmB/PRTM5 and LAP-TDRD6/PRMT5 were performed with the following changes: Single cells were fixed in 1% formaldehyde solution for 5 min and then quenched for 5 min with 200mM Glycine at RT. After the cells were pelleted and lysed in the extraction buffer (1%SDS, 10mM EDTA, 50mM Tris pH8.0, 1mM DTT, 5mM NaF, 1mM Na_2_VO_3_, 1mM PMSF and 1x protease inhibitor cocktail EDTA-free mini), the extracts were diluted at a 1:9 ratio in the IP buffer (0.01%SDS, 1.1% TritonX-100, 1.2mM EDTA, 16.7mM Tris pH8.0, 5mM NaF, 1mM Na_2_VO_3_, 1mM PMSF and 1x protease inhibitor cocktail EDTA-free mini) to quench the excess SDS in the extraction buffer. For elution, beads were boiled in 2x Laemmli buffer first at 70°C for 1h and then at 95°C for 5 min. Densitometric quantification of the blots was performed using Fiji.

### RNA extraction, DNase treatment, reverse transcription (RT), real time PCR

Depending on experiment, total RNA was extracted from either whole testes or MACS-purified hCD4^+^ cells via Trizol (Invitrogen) according to manufacturer’s instructions. Concentration and purity of the RNA samples were determined by UV absorbance measurements using the NanoDrop 2000c Spectrophotometer (Thermo Scientific).

100ng to 1μg of total RNA was treated with RQ1 RNase-Free DNase (Promega) according to manufacturer’s instructions and reverse transcribed into first strand cDNA via SuperScript II reverse transcriptase (Invitrogen) and random primer mix (NEB) according to manufacturer’s instructions.

2μl of the RT reaction was used directly as a template in a total volume of 20μl of real time PCR reaction with Rotor-Gene SYBR Green PCR Kit (Qiagen) according to manufacturer’s instructions. All the primers were used at a concentration of 1μM and are listed in Table 4 in S1 Supplemental Experimental Procedures. Real-time PCR was carried out in qTower 2.0 system (Analytic Jena). Reactions were run in triplicate with an initial activation step for 5mins at 95°C followed by 40 cycles of 5sec at 95°C and 10sec at 60°C. For each reaction, a no-template as well as a no-RT control sample for each condition was also run in parallel. Comparative quantification analyses of PCR products and melting curve analyses were carried out using the qPCRsoft 2.1 software. Expression data were analyzed using the 2^(-ΔΔCt)^ method and normalized to the expression of housekeeping gene *Tbp1* for the variability in RNA levels in each sample.

### RNA immunoprecipitation (RIP)

We performed the same protocol for RIP as in [13] with the exception that 3μg of anti-Y12 antibody (ThermoScientific) (or control mouse IgG (Santa Cruz)) were added to the RIP reaction instead of anti-UPF1 antibody.

### Statistical analysis

All statistical analyses for the immunofluorescence and RIP data were performed with two-tailed, unpaired t-test using the statistics tool of GraphPad Prism (GraphPad Software, Inc.).

### Transcriptome sequencing and bioinformatics analysis

Differential intron usage was assessed using DEXSeq (v1.16.10) R package [53] and differential exon and junction usage was analyzed via R package JunctionSeq (v0.6.16) [54]. For more details, see Supplemental Experimental Procedures.

## Supporting information

S1 Fig Generation and Characterization of LAP-TDRD6 Transgenic Mouse **(A)** Schematic representation of a genomic region that contains TDRD6 (orange) and the cassette for tagging at the N-terminus adapted from [39]. The neomycin-kanamycin resistance gene (neo) is placed inside an artificial intron flanked by loxP sites. EGFP, enhanced green fluorescent protein; gb2, bacterial promoter; P, PreScission cleavage site; pgk, phosphoglycerate kinase (PGK) promoter; S, S-peptide; sa, splice acceptor; sd, splice donor; T, TEV cleavage site. **(B)** Total protein lysates from the testes of an EGFP-positive founder and from an EGFP-negative mouse separated by SDS-PAGE in adjacent lanes and Western blot was performed using α-EGFP and α-TDRD6 IgGs. **(C)** Fluorescent microscope analysis of EGFP-positive adult testis sections. (i) Sections from stage X-XI and (ii) stage I-II- III were stained with DAPI (blue) only. Cells at different developmental stages are indicated with arrows. Spg: Spermatogonium, P: Pachytene, D: diplotene, RS: Round Spermatid, ES: Elongated Spermatid Scale bar: 50μm.
(TIFF)

S2 Fig TDRD6 is not symmetrically dimethylated at arginine residues. α-EGFP IgG was used to immunoprecipitate LAP-TDRD6 from total cell extracts of adult testis cells cultured in the presence of 5′-deoxy-5′-(methylthio)adenosine (MTA) or DMSO (control) for 16h. Precipitates were divided into two and separated in parallel by SDS-PAGE and Western blotting was performed using α-SYM IgG for sDMA and α-EGFP IgG for LAP-TDRD6.
(TIFF)

S3 Fig The absence of RNA is essential for the interaction of TDRD6 and SmB. RNA was extracted from the flow-through of immunoprecipitations in Fig 2B and run on a 1% agarose gel. Mouse rRNA bands are indicated.
(TIFF)

S4 Fig Analysis of early and late apoptotic as well as dead cell populations upon MTA treatment. FACS analysis of propidium iodide (PI) and Annexin V stained EGFP-positive LAP-TDRD6 adult mice after 16 h of MTA versus control DMSO treatment. *Tdrd6*^*-/-*^ mice were used as a control for Annexin V and PI stainings as they show an extensive apoptotic round spermatid cell population (alive: Annexin V^-^ and PI^-^; early apoptotic: Annexin V^+^ and PI^-^; late apoptotic: Annexin V^+^ and PI^+^; dead: Annexin V^-^ and PI^+^).
(TIFF)

S5 Fig Strategy for the isolation of late prophase I cells from *Tdrd6*^*+/-*^ or *Tdrd6*^*-/-*^ mouse testes. **(A)** hCD4-positive single cells isolated from the testes of 20dpp *Tdrd6*^*+/-*^ and *Tdrd6*^*-/-*^ mice were stained with α-SYCP3 to determine the prophase I stage of the cells. All primary spermatocytes were counted and the percentages of diplotene cells, secondary spermatocytes and round spermatids for each genotype were plotted. (n = 100 for each condition, 3 biological replicates, *P* > 0.05) **(B)** Testis sections from 20dpp *Tdrd6*^*+/-*^ and *Tdrd6*^*-/-*^ littermates were stained with anti-cleaved α-PARP1 (red), α-SYCP3 (green) and DAPI (blue). Scale bar: 100μm. **(C)** The number of apoptotic cells per tubule at 20dpp based on the cleaved PARP1 staining in (C) is presented in a scatter plot. (n = 40 for *Tdrd6*^*+/-*^, n = 43 for *Tdrd6*^*-/-*^).
(TIFF)

S6 Fig Immunoblot of a fraction of the α-Y12 pull-down for RIP was run in parallel with SDS-PAGE. Immunoblotting was performed using α-Y12 IgG for SmB. One representative blot of 3 biological repeats is displayed.
(TIFF)

S7 Fig Quality control of the sequencing and primer design for validations. **(A)** Mapping statistics for transcriptome analysis. Bar plots show the total number of reads (red bars), mapped reads (green bars) which aligned to the reference for each sample and unique reads (blue bars) which aligned uniquely to the reference for each sample. **(B)** Schematic representation of the primer design for the validation of the differential usage of I1 of *Efhb*. The 5’ end lies on the left hand-side. Intron and exons with differential usage are shown in magenta. I, intron; E, exon; Fwd, forward primer; Rev, reverse primer.
(TIFF)

S1 Supplemental Experimental Procedures (PDF)
