# Correction: TDRD6 mediates early steps of spliceosome maturation in primary spermatocytes

**DOI:** 10.1371/journal.pgen.1006989

**Published:** 2017-09-01

**Authors:** Müge Akpınar, Mathias Lesche, Grigorios Fanourgakis, Jun Fu, Konstantinos Anastassiadis, Andreas Dahl, Rolf Jessberger

The fifth author’s name is spelled incorrectly. The correct name is Konstantinos Anastassiadis: The correct citation is: Akpınar M, Lesche M, Fanourgakis G, Fu J, Anastassiadis K, Dahl A, et al. (2017) TDRD6 mediates early steps of spliceosome maturation in primary spermatocytes. PLoS Genet 13(3): e1006660. https://doi.org/10.1371/journal.pgen.1006660
