# Supplementary figures and images for: TDRD6 mediates early steps of spliceosome maturation in primary spermatocytes
Source: PLoS Genet. 2017 Mar 6;13(3):e1006660. doi: 10.1371/journal.pgen.1006660 (PMC5358835; doi:10.1371/journal.pgen.1006660)

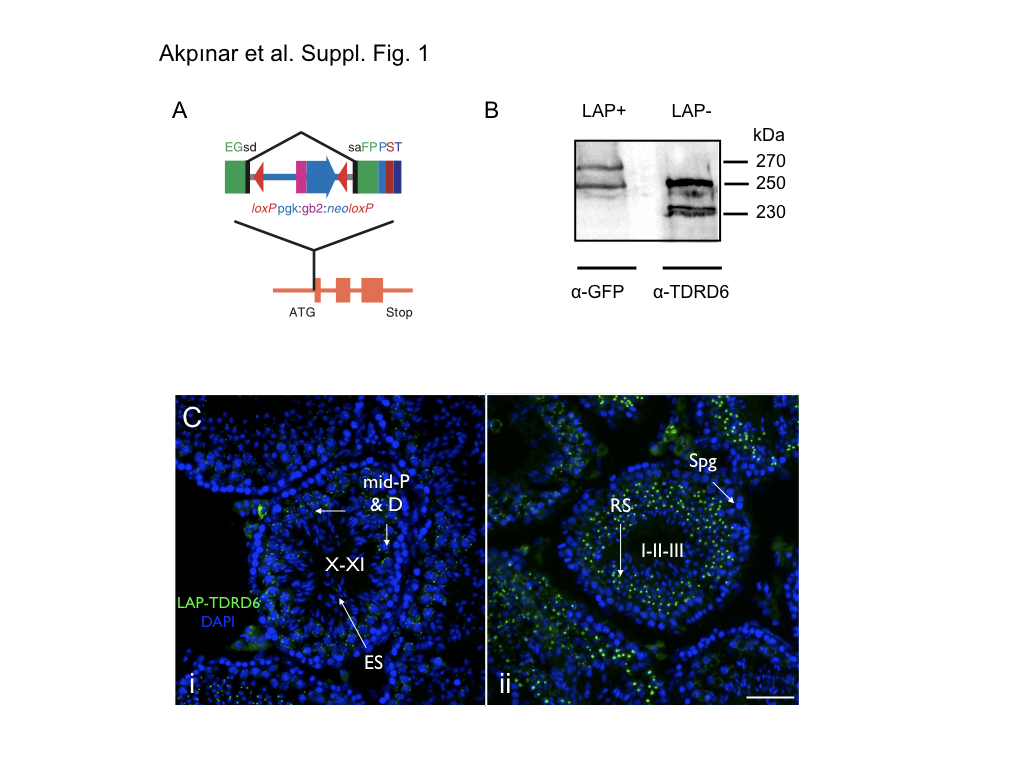

Supplement: S1 Fig — (A) Schematic representation of a genomic region that contains TDRD6 (orange) and the cassette for tagging at the N-terminus adapted from [39]. The neomycin-kanamycin resistance gene (neo) is placed inside an artificial intron flanked by loxP sites. EGFP, enhanced green fluorescent protein; gb2, bacterial promoter; P, PreScission cleavage site; pgk, phosphoglycerate kinase (PGK) promoter; S, S-peptide; sa, splice acceptor; sd, splice donor; T, TEV cleavage site. (B) Total protein lysates from the testes of an EGFP-positive founder and from an EGFP-negative mouse separated by SDS-PAGE in adjacent lanes and Western blot was performed using α-EGFP and α-TDRD6 IgGs. (C) Fluorescent microscope analysis of EGFP-positive adult testis sections. (i) Sections from stage X-XI and (ii) stage I-II- III were stained with DAPI (blue) only. Cells at different developmental stages are indicated with arrows. Spg: Spermatogonium, P: Pachytene, D: diplotene, RS: Round Spermatid, ES: Elongated Spermatid Scale bar: 50μm. (TIFF) [file pgen.1006660.s001.tiff]

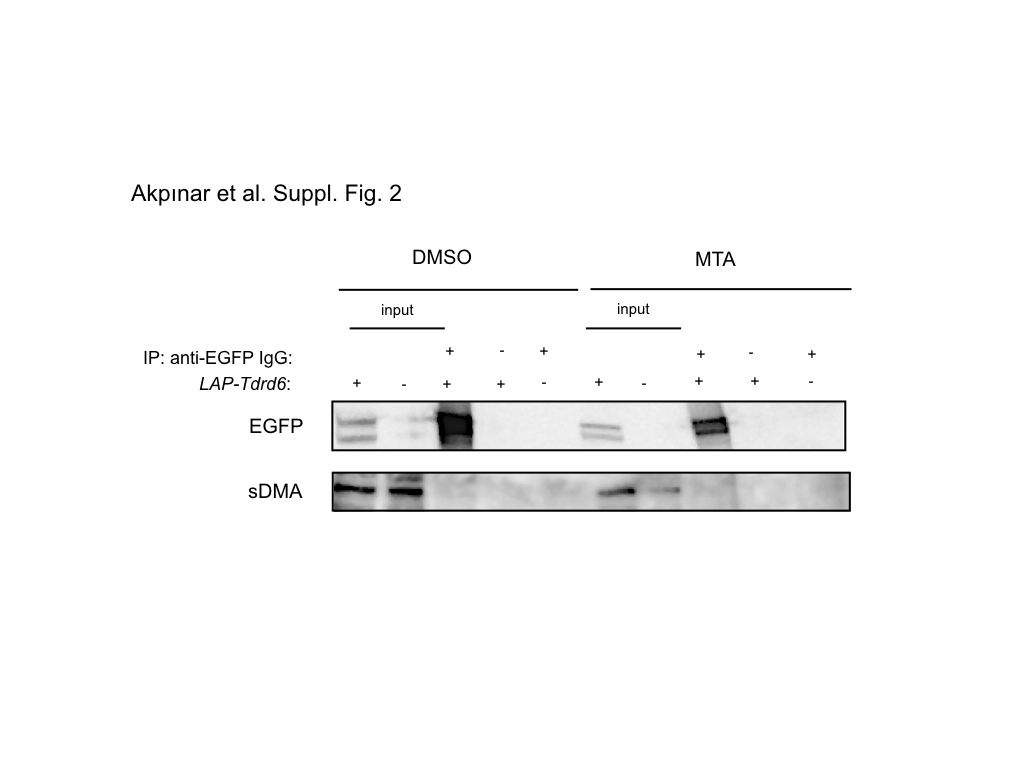

Supplement: S2 Fig — α-EGFP IgG was used to immunoprecipitate LAP-TDRD6 from total cell extracts of adult testis cells cultured in the presence of 5′-deoxy-5′-(methylthio)adenosine (MTA) or DMSO (control) for 16h. Precipitates were divided into two and separated in parallel by SDS-PAGE and Western blotting was performed using α-SYM IgG for sDMA and α-EGFP IgG for LAP-TDRD6. (TIFF) [file pgen.1006660.s002.tiff]

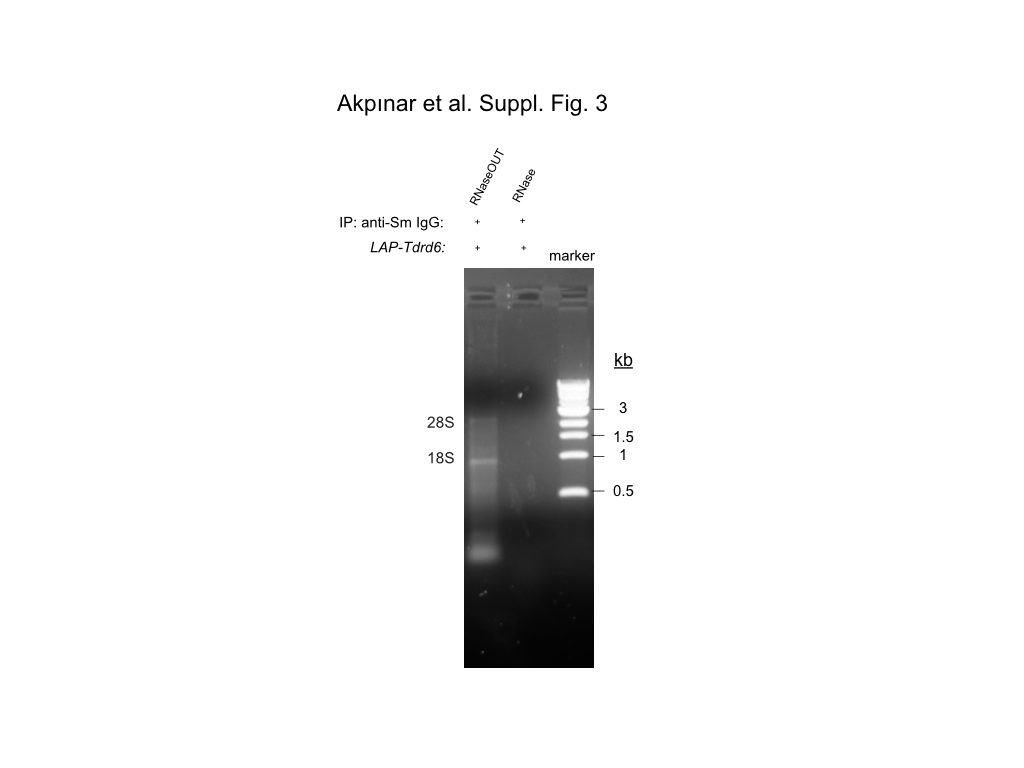

Supplement: S3 Fig — RNA was extracted from the flow-through of immunoprecipitations in Fig 2B and run on a 1% agarose gel. Mouse rRNA bands are indicated. (TIFF) [file pgen.1006660.s003.tiff]

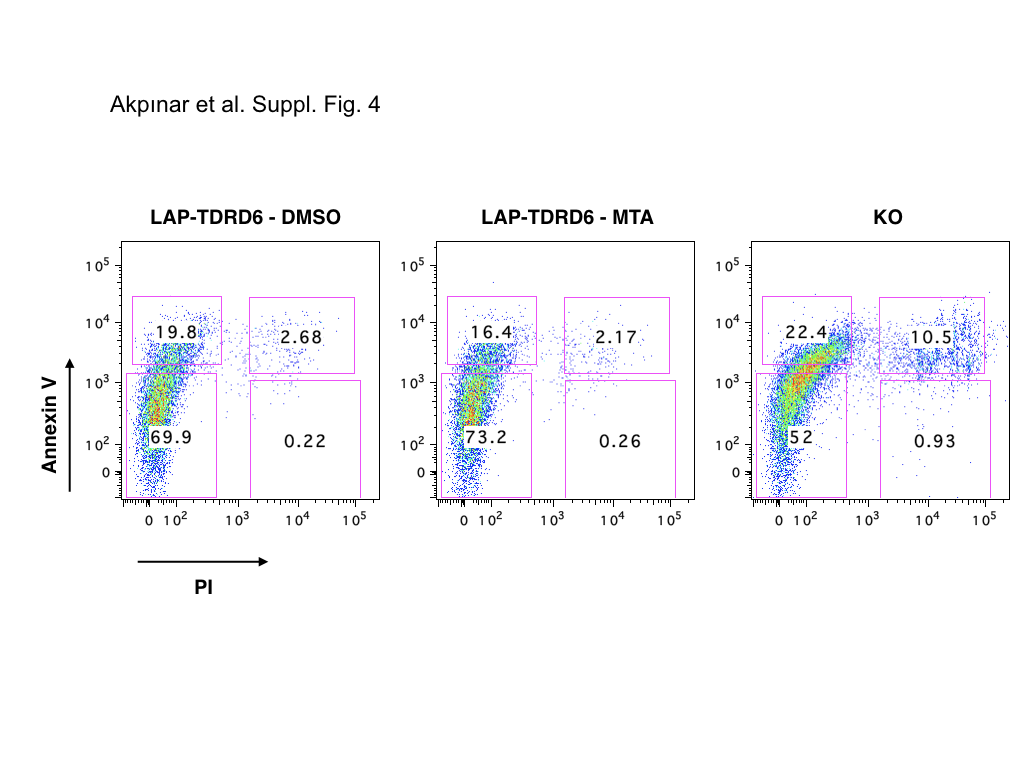

Supplement: S4 Fig — FACS analysis of propidium iodide (PI) and Annexin V stained EGFP-positive LAP-TDRD6 adult mice after 16 h of MTA versus control DMSO treatment. Tdrd6-/- mice were used as a control for Annexin V and PI stainings as they show an extensive apoptotic round spermatid cell population (alive: Annexin V- and PI-; early apoptotic: Annexin V+ and PI-; late apoptotic: Annexin V+ and PI+; dead: Annexin V- and PI+). (TIFF) [file pgen.1006660.s004.tiff]

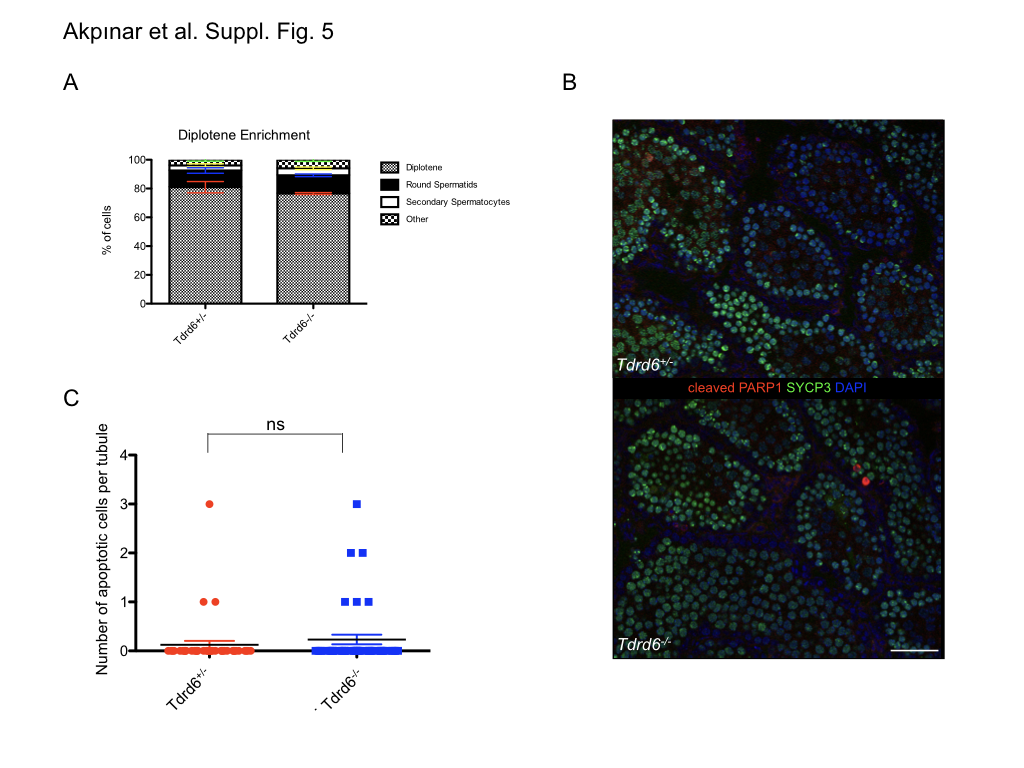

Supplement: S5 Fig — (A) hCD4-positive single cells isolated from the testes of 20dpp Tdrd6+/- and Tdrd6-/- mice were stained with α-SYCP3 to determine the prophase I stage of the cells. All primary spermatocytes were counted and the percentages of diplotene cells, secondary spermatocytes and round spermatids for each genotype were plotted. (n = 100 for each condition, 3 biological replicates, P > 0.05) (B) Testis sections from 20dpp Tdrd6+/- and Tdrd6-/- littermates were stained with anti-cleaved α-PARP1 (red), α-SYCP3 (green) and DAPI (blue). Scale bar: 100μm. (C) The number of apoptotic cells per tubule at 20dpp based on the cleaved PARP1 staining in (C) is presented in a scatter plot. (n = 40 for Tdrd6+/-, n = 43 for Tdrd6-/-). (TIFF) [file pgen.1006660.s005.tiff]

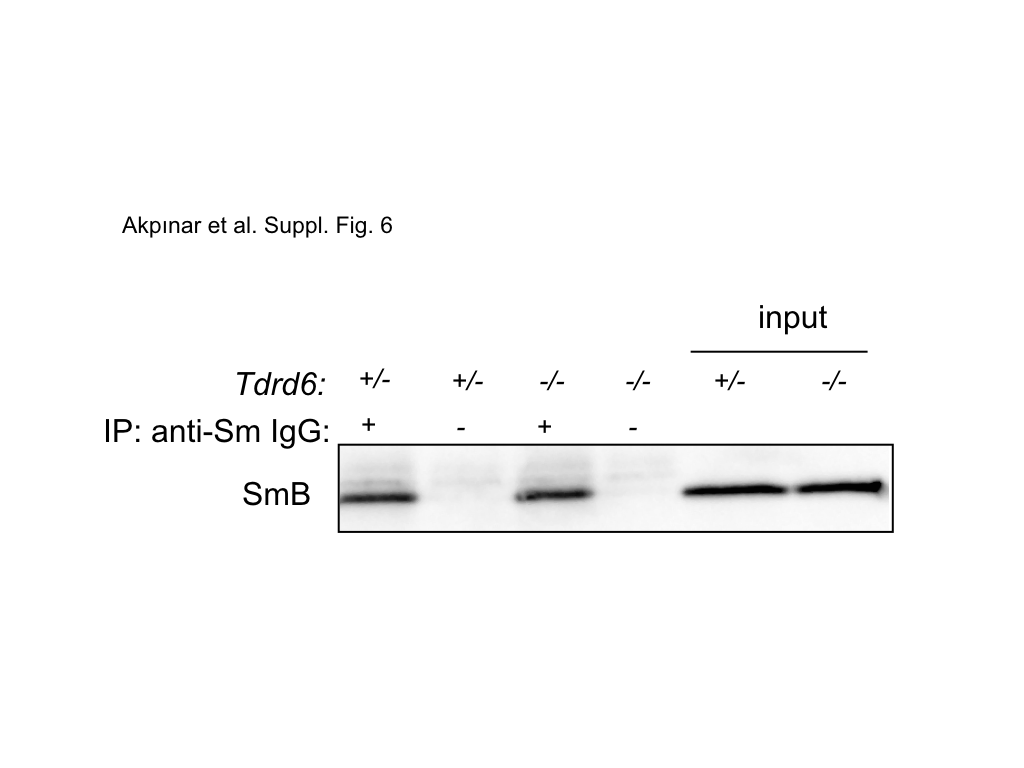

Supplement: S6 Fig — Immunoblotting was performed using α-Y12 IgG for SmB. One representative blot of 3 biological repeats is displayed. (TIFF) [file pgen.1006660.s006.tiff]

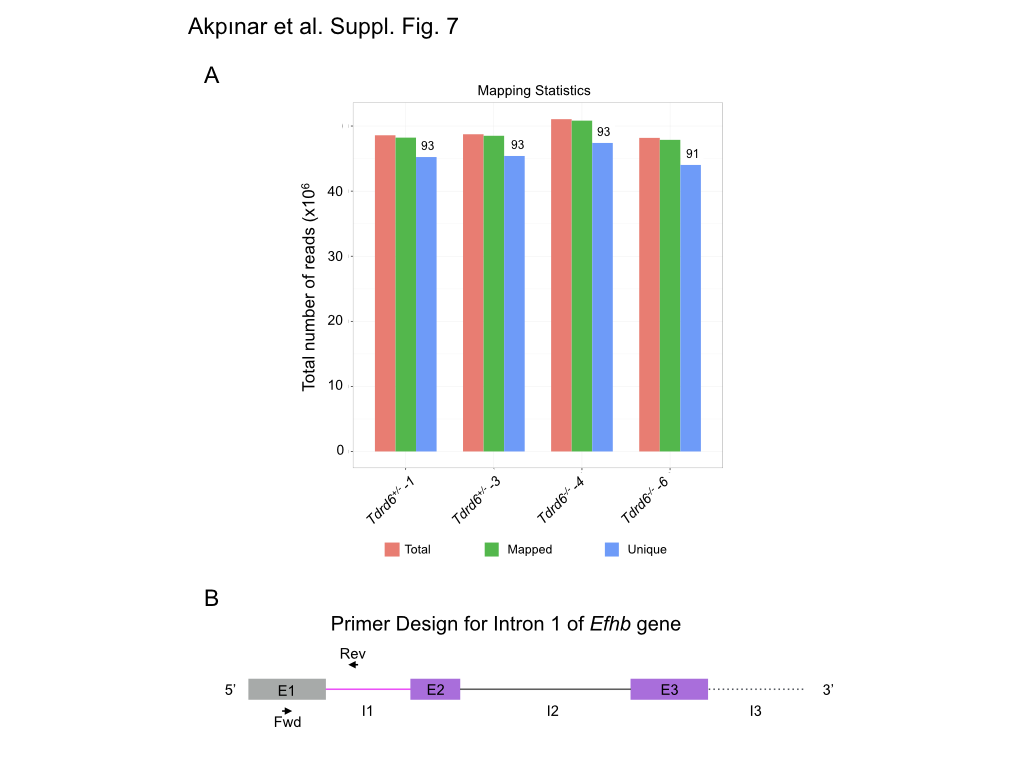

Supplement: S7 Fig — (A) Mapping statistics for transcriptome analysis. Bar plots show the total number of reads (red bars), mapped reads (green bars) which aligned to the reference for each sample and unique reads (blue bars) which aligned uniquely to the reference for each sample. (B) Schematic representation of the primer design for the validation of the differential usage of I1 of Efhb. The 5’ end lies on the left hand-side. Intron and exons with differential usage are shown in magenta. I, intron; E, exon; Fwd, forward primer; Rev, reverse primer. (TIFF) [file pgen.1006660.s007.tiff]
