# Supplementary material for: TDRD6 mediates early steps of spliceosome maturation in primary spermatocytes
Source: PLoS Genet. 2017 Mar 6;13(3):e1006660. doi: 10.1371/journal.pgen.1006660 (PMC5358835; doi:10.1371/journal.pgen.1006660)
Supplement: S1 Supplemental Experimental Procedures — (PDF) [file pgen.1006660.s008.pdf]

Supplemental Experimental Procedures

Mouse Strains

Tdrd6$^{-/-}$ mice have been previously generated by our group, characterized and described in detail [1]. Since *Tdrd6*$^{+/-}$ mice are phenotypically the same as wild type mice, we used them as controls for their *Tdrd6*$^{-/-}$ littermates.

The *Tdrd6-LAP* construct was generated by recombineering [2] using the BAC clone RP23-283P8 carrying the locus (ENSMUSG00000040140) of *Tdrd6* on 17th chromosome of *Mus musculus* genome. The recombination construct carries the EGFP sequence preceded by the *Tdrd6* promoter to generate an N-terminally tagged *Tdrd6* gene within the endogenous gene expression control elements. For making the modified BAC transposition compatible, a cassette carrying the piggyBac inverted repeats flanking the ampicillin gene was inserted in the bacterial backbone. The Tdrd6-LAP BAC was injected into pronucleus of C57BL/6 mice together with a piggyBac transposase mRNA by the Transgenic Core Facility of the MPI-CBG, Dresden, as previously described [3]. Injection of a circular BAC together with the transposase resulted in total transgenesis frequency of 13% (132 injected oocytes/24 born pups/17 transgenic and 15 of them had transposition signature), whereas transgenesis ratio after injection of a linear BAC was 2% (447 injected/54 born/10 transgenic). Three founders were mated with *Tdrd6*$^{-/-}$ mutants. Genotyping of the transgenic mice was performed using EGFP primers listed in Table 2.4. Mice were maintained and bred in the Experimental Animal Center of the Medical Theoretical Center of Dresden University of Technology under pathogen-free conditions. All mice were housed, bred and sacrificed

according to institutional guidelines.

Apoptosis Assay via Fluorescent Activated Cell Sorting (FACS)

Single cell suspension of the total testis from LAP-TDRD6 mice was obtained by removing the *tunica albuginea*, placing the seminiferous tubules into cold PBS on ice and passing the cells through 100μm and 40μm cell sieves subsequently. After culturing of the cells with MTA or DMSO for 16 h, cells were harvested, counted and 2 million cells were stained with 0.8 μg/ml of Pacific Blue™-labeled anti-Annexin-V antibody (Biolegend; Cat No: 640918) in 200 μl Annexin V binding buffer (10mM HEPES, 140 mM NaCl and 2.5mM $CaCl_2$, pH 7.4) for 30 min at room temperature in dark. Cell volume was increased to 500 μl in the same buffer, supplemented with 2 μg/ml propidium iodide and immediately analyzed on a BD LSRII (BD Biosciences) using FACSDiva software (BD Biosciences). Data were analyzed using FlowJo software (TreeStar).

Primary Spermatocyte Isolation via MACS

Tdrd6$^{-/-}$ mice have the first exon of *Tdrd6* locus replaced with the truncated human CD4 gene deficient for intracellular signaling yet still capable of translocating to the cellular membrane [1]. First, the single cell suspension of the total testis was obtained by removing the *tunica albuginea*, placing the seminiferous tubules into cold PBS on ice and passing the cells through 100μm and 40μm cell sieves subsequently. Since hCD4 expression is driven by the endogenous Tdrd6 promoter, Tdrd6 expressing germ cells (i.e. late-pachytene cells and beyond) of males carrying at least one allele of the knocked out *Tdrd6* locus (*Tdrd6*$^{+/-}$ or *Tdrd6*$^{-/-}$) are isolated using anti-hCD4

antibody coupled magnetic MACS beads (Miltenyi Biotec) according to manufacturer's instructions.

Protein Extracts, Western blotting

All the steps were performed at 4°C unless otherwise indicated.

For total cell extracts, single cells were resuspended in RIPA buffer (50mM Tris-HCl at pH 7.4, 150mM NaCl, 1% NP40, 0.5% sodiumdeoxycholate, 0.1%SDS, 1mM DTT, 1mM EDTA, 5mM NaF, 1mM Na_2VO_3, 1mM PMSF, 1x protease inhibitor cocktail EDTA-free mini), incubated for 20mins, sonicated twice for 15sec with 1min interval. Cell debris was precipitated by centrifugation at 13000rpm for 10mins.

The protein concentration in the supernatant was quantified via Roti-Quant kit according to manufacturer's instructions. 50µg protein was resolved in 4–20% gradient precast polyacrylamide gel (BioRad) under constant current of 25mA/gel at room temperature. Proteins were then transferred under constant voltage of 100 V for 2 hours to Hybond ECL membrane, which is then blocked for 1h at room temperature in 5% milk/0.1% Tween-20/PBS, incubated with the primary antibody diluted in 0.1% Tween-20/PBS overnight. Next day the membrane was washed 3 times for 10mins in 0.1% Tween-20/PBS and incubated with the secondary antibody (1:10000) in the same wash buffer for 1h at room temperature. After the final wash, Immobilon Western HRP Substrate detection reagent was applied onto the membrane and the luminescence was immediately detected via the KODAK Image Station 2000MM.

RNA Isolation

Wherever indicated, RNA was isolated from the flow-through of the IPs with Trizol after overnight incubation as described below and RNA degradation or integrity upon RNase or RNaseOUT treatment was assessed by running the RNA samples in 1% agarose gels and checking the mouse rRNA bands.

The quality of the RNA samples for deep sequencing was additionally checked by RNA integrity (RIN) analysis at the DNA Microarray Facility in Max Planck of Molecular Cell Biology and Genetics via Agilent 2100 Bioanalyzer. Samples with a RIN number of 8.5 to 9.1 were used for sequencing.

Immunostainings for Testicular Sections

After surgical removal, testes were fixed using 4% formaldehyde (Sigma) and 0.15% Triton X-100 in PBS for 30 min at room temperature and later placed into 30% sucrose/PBS for overnight incubation. Next day, they were embedded in Tissue TEK O.C.T.^{TM} (Weckert Labortechnik), shock-frozen on dry ice and stored at -80°C. After at least one day, 7µm sections were cut with Leica CM1900 Cryostat, placed on object slides, re-fixed in 2% formaldehyde/PBS for 5min at room temperature, washed once with PBS, incubated in permeabilization/blocking buffer (0.15% bovine serum albumin, 0.1% Tween-20) for 1h at room temperature and incubated overnight with the primary antibody in the same permeabilization/blocking buffer at 4°C. After three washes with PBS-0.1% Tween-20 each for 5min, they were incubated with the secondary antibody in permeabilization/blocking buffer for 1h at room temperature, washed again as before, mounted in Fluoromount-G (Biozol)

with 0.25µg/ml DAPI for nuclear visualization and covered with a coverslip. Stainings were visualized using the Leica SP 5 confocal laser-scanning microscope and the images were analyzed with the softwares LAS AF and Fiji.

Transcriptome Sequencing

a. Library preparation and sequencing

Library preparation and subsequent sequencing were performed by the Deep Sequencing Group of Dr Andreas Dahl in BIOTEC, TU Dresden. Genomic DNA was removed from the total RNA with DNase I, followed by rRNA depletion using the Ribo-Zero™ Magnetic Gold Kit from Epicentre. Libraries were prepared by following the protocol for NEBNext® Ultra Directional RNA Library Prep Kit: RNA fragmentation, first strand and second strand cDNA synthesis, purification using the Agencourt® AMPure® Kit and end repair/dA-tailing of cDNA. Adapters were ligated to the dA-tailed cDNA, followed by a size selection using AMPure XP Beads. Indexing of the library constructs was done with Illumina® index primer during the PCR amplification using NEBNext® High-Fidelity 2X PCR Master Mix. Finally, libraries were purified using the Agencourt® AMPure® Kit. Libraries were pooled and sequenced on an Illumina® HiSeq 2500, resulting in ca. 47-51 million paired-end fragments. This experiment was performed in duplicates (two controls, two mutants), each of which is derived by pooling at least 4 different animals.

b. Bioinformatics Analysis

Alignment and bioinformatics analysis were performed by Mathias Lesche (Deep Sequencing Group, BIOTEC, TU Dresden).

c. Alignment and Quality

FastQC (http://www.bioinformatics.babraham.ac.uk/) and RNA-SeQC (v1.1.8) were used to perform a basic quality control on the sequenced fragments. Alignment of the fragments to mouse reference genome (release mm10, December 2011) was done with GSNAP (v2014-12-17) [43]. Ensembl annotation 75 (release February 2014) [4] was used to detect fragments spanning splice sites.

d. Differential intron usage

In order to perform a differential intron usage analysis, one requires an annotation file that lists the intronic regions for each gene and its isoforms. This file was created manually by using the standard Ensembl annotation file, which was used for alignment and also for fragment counting. The Ensembl file lists many exons multiple times, for each isoform that contains it once. This redundant information was collapsed and exon bins were defined. Each exon bin represents the isoforms to which it originally belongs. Exons that originated from more than one gene were removed because it would not be possible to clearly assign fragments for them. A script (dexseq_prepare_annotation.py) from the DEXSeq (v1.16.10) R package was used for this step. The bin between two exon bins is assigned as an intron region. These intron bins were built with a custom script and the modified annotation file. Genes were removed which only had a single intron because at least two introns per gene are needed to test for differential intron usage. Fragments overlapping these intron bins were counted with another script (dexseq_count.py) from the DEXSeq package and used for differential intron usage testing with DEXSeq, which was originally developed for differential

exon usage detection but it is possible to use it for introns as well because the underlying methodology does not change. *Tdrd6*$^{+/-}$ was compared to *Tdrd6*$^{-/-}$, accepting a maximum of 10% false discovery rate ($p_{adj} < 0.1$). The analysis fits a generalized linear model with the formula sample + intron + condition:intron and compares it to the null model sample + intron.

e. Differential exon and splice site usage

The procedure for the detection of differential exon and splice site usage is similar compared to the differential intron usage detection. QoRTs (v1.0.1) was used to generate a collapsed annotation file from the original Ensembl annotation. The R package JunctionSeq (v0.6.16) [5] was used for the actual testing. The program methodology is heavily based on DEXSeq but offers the possibility to check for splice sites as well.

Tables

Table 1. Antibodies used for Immunoblotting

Antibody	Host	Conc./Dil.	Source
α-EGFP, polyclonal	goat	1μg/ml	Hyman lab, MPI-CBG Dresden
α-PRMT5 (07-405), polyclonal	rabbit	2μg/ml	EMD Millipore
α-dimethyl-Arginine, symmetric (SYM10), polyclonal	mouse	2μg/ml	Merck Millipore
α-Sm (Y12, MS450), monoclonal	mouse	0.4μg/ml	Thermo Scientific
α-TDRD6 (AAS11661C), polyclonal	rabbit	2μg/ml	Antibody Verify

Table 2. Antibodies used for Immunoprecipitations

Antibody	Host	Conc./Dil.	Source
α-EGFP, polyclonal	goat	1μg	Hyman lab, MPI-CBG Dresden
α-PRMT5 (07-405), polyclonal	rabbit	1.5μg	EMD Millipore
α-Sm (Y12,MS450), monoclonal	mouse	2μg	Thermo Scientific

Table 3. Antibodies used for Immunostainings

Antibody	Host	Conc./Dil.	Source
α-SYCP3 (monoclonal hybridoma supernatant, clone 60C10), IgG2a	mouse	1:1	In house
α-dimethyl-Arginine, symmetric (SYM10), polyclonal	rabbit	1:500	Merck Millipore
α-SMN (2B1; NB100-1936), monoclonal, IgG1	mouse	1:500	Novus
α-sm (Y12, MS450), monoclonal, IgG3	mouse	1:100	Thermo Scientific
α-cleaved PARP1 (#9544), monoclonal	rabbit	1:500	Cell Signaling
α-MVH (ab13840), polyclonal	rabbit	1:500	Abcam
α-coilin (H-300), polyclonal	rabbit	1:200	Santa Cruz

Table 4 Primers used in this study

Gene Symbol	Primer	Sequence	Source
U2	F<br>R	5’-ATCGCTTCTCGGCCTTTTG-3’<br>5’-TCCTGGAGGTACTGCAATACC-3’	Eurofins Genomics
U5	F<br>R	5’-GGTTTCTCTTCAGATCGTATA-3’<br>5’-TTGGTTTAAGACTCAGAGTT-3’	Eurofins Genomics
U12	F<br>R	5’-ATAACGATTCGGGGTGACGC-3’<br>5’-TGCAACCTTTACCCGCTCAA-3’	Eurofins Genomics
U4atac	F<br>R	5’-CGCTACTGTCCAATGAACGC-3’<br>5’-AGCAGAGCTCTAACCGATGC-3’	Eurofins Genomics
Akt3 I7	F<br>R	5’-GTCTTAGAGTGCTCAGTGTCC-3’<br>5’-CCATAGAAACGTGTGCGGTC-3’	Eurofins Genomics
Cage1 I1	F<br>R	5’-CTTGTGTATGCGTGCTCCGT-3’<br>5’-TGAACAGGAAAAAGCGCGGA-3’	Eurofins Genomics
Cers3 I10	F<br>R	5’-TTCAGGGCCTCCACGTTTAC-3’<br>5’-TGCTGTCTGAACCTCGCTTT-3’	Eurofins Genomics
Efhb I1	F<br>R	5’-GCCCAACAACCGGAAGAGAA-3’<br>5’-CACGAGTTCCAGGGGAAACC-3’	Eurofins Genomics
Nipbl I34	F<br>R	5’-GCCATGACTATGCAGCCGTA-3’<br>5’-GGCACTTACTGCCTGTTGC-3’	Eurofins Genomics
Stim1 I7	F<br>R	5’-AGCACCGAACTGTGGAAGTAG-3’<br>5’-CTGATCCTCTGCCCTTTACGC-3’	Eurofins Genomics
Tbp1	F<br>R	5’-GCAGTGCCCAGCATCACTAT-3’<br>5’-TGGAAGGCTGTTGTTCTGGT-3’	Eurofins Genomics
EGFP	F<br>R	5' -CTTCTTCAAGGACGACGGCAACTA-3'<br>5' -ATCGCGCTTCTCGTTGGGGTCTTTGC- 3'	Eurofins Genomics

References for Supplemental Information

1. Vasileva A, Tiedau D, Firooznia A, Muller-Reichert T, Jessberger R. Tdrd6 is required for spermiogenesis, chromatoid body architecture, and regulation of miRNA expression. Curr Biol. 2009;19(8):630-9. Epub 2009/04/07. doi: S0960-9822(09)00757-X [pii]
10.1016/j.cub.2009.02.047. PubMed PMID: 19345099.
2. Poser I, Sarov M, Hutchins JR, Heriche JK, Toyoda Y, Pozniakovsky A, et al. BAC TransgeneOmics: a high-throughput method for exploration of protein function in mammals. Nat Methods. 2008;5(5):409-15. Epub 2008/04/09. doi: nmeth.1199 [pii]
10.1038/nmeth.1199. PubMed PMID: 18391959.
3. Rostovskaya M, Naumann R, Fu J, Obst M, Mueller D, Stewart AF, et al. Transposon mediated BAC transgenesis via pronuclear injection of mouse zygotes. Genesis. 2013;51(2):135-41. Epub 2012/12/12. doi: 10.1002/dvg.22362. PubMed PMID: 23225373.
4. Flicek P, Amode MR, Barrell D, Beal K, Billis K, Brent S, et al. Ensembl 2014. Nucleic Acids Res. 2014;42(Database issue):D749-55. Epub 2013/12/10. doi: gkt1196 [pii]
10.1093/nar/gkt1196. PubMed PMID: 24316576; PubMed Central PMCID: PMC3964975.
5. Hartley SW, Mullikin JC. Detection and visualization of differential splicing in RNA-Seq data with JunctionSeq. Nucleic Acids Res. 2016;44(15):e127. Epub 2016/06/04. doi: gkw501 [pii]
10.1093/nar/gkw501. PubMed PMID: 27257077; PubMed Central PMCID: PMC5009739.
